# Tooth and temporary filling material fractures caused by Cavit, Cavit W and Coltosol F: an in vitro study

**DOI:** 10.1186/s12903-021-01431-4

**Published:** 2021-02-16

**Authors:** Bedram Djouiai, Thomas Gerhard Wolf

**Affiliations:** 1 Department of Restorative, Preventive and Pediatric Dentistry, School of Dental Medicine, University of Bern, Freiburgstrasse 7, 3010 Bern, Switzerland; 2 Department of Periodontology and Operative Dentistry, University Medical Center of the Johannes Gutenberg-University Mainz, Mainz, Germany

**Keywords:** Cavit, Cavit W, Coltosol, Endodontic treatment, Fracture, Temporary filling material, Tooth fracture

## Abstract

**Background:**

Tooth fractures can occur after temporary inter-appointment endodontic filling, resulting in not preserving and thus extraction of the affected tooth. The purpose of this investigation was therefore to evaluate the tooth substance fracture potential given by the expansion of endodontic temporary filling materials.

**Methods:**

Tooth and access cavities were prepared in 80 mandibular molars. Four groups of 20 teeth each (Cavit, Cavit W and Coltosol F and control) were included. To simulate a clinical situation, the teeth were endodontically pre-treated and a calcium hydroxide dressing was placed. The cavities were filled with the corresponding temporary filling material, with exception of the control group, and kept submerged in distilled water for 15 days. The teeth were examined every 24 h by two calibrated observers under a stereomicroscope (7.5×), fractures of the temporary filling material and tooth structure were photo-documented, and the results statistically analyzed. Kaplan–Meier survival analysis were calculated to illustrate (survival = no fracture) probabilities to evaluate the time when the temporary filling material, tooth structure or both together occurred. Log-rank test was performed in order to assess significant differences between the materials and the subgroups used.

**Results:**

Fractures were observed only in the Coltosol F group (*p* < 0.01), at the end of the observation period, a total of 13 teeth (65%) showed temporary filling material and eight teeth (40%) showed tooth structure fractures. No fractures in the pulp chamber area were observed at the end of the observation period in any group.

**Conclusions:**

Within the limitations of the current in vitro study, the results obtained suggest that tooth structure fractures caused by a temporary filling material can occur during endodontic treatment, thus compromising the success of the treatment.

## Background

Endodontic treatment is one of the most frequently applied treatment modalities in the dental clinical routine and can be caused by various conditions such as bactericidal infections, trauma and iatrogenic factors [1, 2]. Single and multi-endodontic visit treatments have often served as a focus of controversy in literature [3, 4]. Even if there is no solid evidence that one treatment modality is superior to the other [4], it has been suggested that a multi-visit approach may be preferred for certain pulpal condition [3]. Elimination of bacteria and pulp tissue through chemical and mechanical debridement and a hermetic root canal system obturation are the key to endodontic treatment success [5]. Along with these steps, during a multi-visit treatment, an adequate inter-appointment access cavity closure through a temporary filling also has a significant impact on the outcome of a root canal treatment [6]. Therefore, a temporary inter-appointment endodontic filling must fulfill certain requirements, such as prevention of bacterial pulp space contamination and root canal dressing or saliva leakage [7]. Furthermore, since a tooth structure fracture is one of the major concerns which can lead to tooth loss, an adequate prevention against the development of cracks and/or fractures of the tooth structure during as well as after endodontic treatment is mandatory [8, 9]. Tooth fractures can occur during an endodontic treatment due to the inherent tooth structure loss [10] and, eventually, through stress produced by the coronal temporary filling material [11, 12].

The main purpose of an endodontically temporary filling material is the maintenance of a coronal hermetic tooth seal while undergoing an endodontic treatment [7]; however, tooth structure fracture prevention is of no less importance and, thus, also a decisive requirement for temporary fillings as well. Different studies [12–14] have evaluated the effect of different temporary filling materials on tooth structures and have shown that Coltosol F can cause tooth structure fractures; therefore, it has been reported as a disadvantageous temporary filling material for access cavities during an endodontic treatment. Tennert et al. [12] reported that zinc oxide and zinc sulfate-based materials, such as Coltosol F, lead to a 76% tooth structure fracture when used in Class II (OD) cavities in mandibular and maxillary molars. The authors are of the opinion that the fractures observed were caused by the hygroscopic tendency of this material. Laustsen et al. [14] also reported vertical tooth fractures in 44% of Class II (MOD) cavities in molars also sealed with Coltosol F after an observation period of 20 days. In contrast, Balkaya et al. [13] investigated Class II (MOD) cavities. However, of maxillary premolars restored with zinc oxide and zinc sulfate-based (Coltosol F and Cavit G), polymer-reinforced zinc oxide-eugenol based and a light-curing silver containing materials and reported that it is rather the cavity design than the temporary filling material that have an impact on tooth structure fracture resistance.

Cavit (pink), Cavit W (white) and Coltosol F are three widely spread inter-appointment temporary filling materials for access cavities during endodontic treatment [15]. Cavit is manufactured in three different modalities: Cavit (pink), Cavit W (white) and Cavit G (grey) and are intended for different clinical purposes. The exact composition of Cavit, Cavit W and Coltosol F is not provided by the manufacturers. Cavit and Cavit W are materials containing zinc oxide and zinc sulfate in different concentrations with varying additives, which, according to the manufacturer, results in a different final hardness for Cavit and in an adhesion increase for Cavit W [16, 17]. Coltosol F is also a zinc oxide and zinc sulfate-based temporary filling material for Class I and II cavities [18]. Since Cavit, Cavit W and Coltosol F are temporary filling materials containing zinc oxide and zinc sulfate, it can be assumed that these temporary filling materials would have similar cavity sealing and stress characteristics. Thus, the aim of this in vitro research was to investigate the tooth and temporary filling material fracture potential of these three inter-appointment temporary filling materials in mandibular molars during endodontic treatment.

## Methods

### Tooth selection

Permanent human two-rooted mandibular molars were collected from dentists and dental clinics in Switzerland for reasons not concerning this investigation and stored in a 3% chloramine solution. The teeth were anonymized with personal data not traceable; therefore, an ethical approval was not required. All selected teeth could be clearly identified as mandibular molars by two independent reviewers [19, 20]. The teeth selection was made under a stereomicroscope (7.5× magnification; Leica M420, Leica Microsystems, Wetzlar, Germany). The selection criteria were no signs of fractures, restorations, caries or endodontic treatment and no mandibular third molars. All remnants were removed from the teeth external surfaces with a universal curette (Hu Friedy, Rotterdam, The Netherlands) and a prophylaxis brush with plastic bristles (KaVo Kerr, Brea CA, USA).

### Cavity preparation

The clinical condition of an inter-appointment situation of 80 mandibular molars during endodontic treatment was simulated through the preparation of a standardized mesio-occlusal cavity derived from the study of Tennert et al. [12] with a pear-shaped red diamond and a corresponding finisher (yellow) size 2 bur (Gebr. Brasseler GmbH and Co KG, Lemgo, Germany). The occlusal cavity isthmus had a width of 3 mm (± 0.4 mm), a height of at least 2 mm and a minimal residual dentin thickness from the access cavity mesial limit to the approximal surface of 1.5 mm (± 0.4 mm). The approximal surface had a width and height of 4 mm (± 0.4 mm), a gingival wall depth of 2 mm (± 0.4 mm), an axial wall height of 1.5–2 mm (± 0.4 mm) and a distance from its gingival limits to the cement-enamel junction of 1 mm (± 0.4 mm).

### Access cavity and chemo‐mechanical preparation

The access cavities were prepared by removing the pulp chamber roof with a diamond pear-shaped red size 2 bur (Komet, Lemgo, Germany) and shaped with an Endo-Z bur (Dentsply Maillefer, Tulsa, USA). After pathfinding the root canals with an ISO 06 C-Pilot file (VDW, Munich, Germany), the working length was determined with a ISO 10 K-type file (VDW, Munich, Germany) which was inserted into the root canal and guided gently in the apical direction until its tip appeared, under magnification (7.5×), at the physiological foramen limits. The root canals were then prepared sequentially as recommended by the manufacturer by means of a WaveOne Gold Glider 15/.02, WaveOne Gold Small 20/.07, Primary (25/.07) to a Medium (35/.06) size file reciprocating system (Dentsply Sirona, Ballaigues, Switzerland) and endodontic motor (X-Smart Plus/Dentsply Sirona, Ballaigues, Switzerland) with an alternating irrigation of a 3% NaOCl solution, and 17% EDTA [21].

### Apical region sealing and root canal dressing

The teeth were kept immersed in water at 37 °C at all times. In order to avoid the penetration of water into the root canal system, the apical 5 mm of the roots were sealed with Ceram X mono composite (Dentsply Sirona, Ballaigues, Switzerland) with incremental technology, as recommended by the manufacturer, directly applied from the compule and adapted in the apical region with a dental spatula. During this procedure, a corresponding gutta-percha cone (VDW, Munich, Germany) was inserted into each root canal to prevent the adhesive chemicals or composite from penetrating into the root canal system. Before sealing with the mono composite used, conditioning of the root surfaces was made with OptiBond FL (KaVo Kerr, Brea CA, USA) and the composite light-cured for 40 s (Elipar FreeLight 2/3 M ESPE, St. Paul, MN, USA). The gutta-percha cones were removed afterwards. Since a parameter aim of this investigation was to simulate a clinical situation, a calcium hydroxide (Calcicur/Voco, Cuxhaven, Germany) dressing was placed into the root canals with a ISO 15 K-type file (VDW, Munich, Germany). Excess material on the pulp chamber walls and floor was removed with a dry foam pellet.

### Temporary restoration

**Fig. 1:**
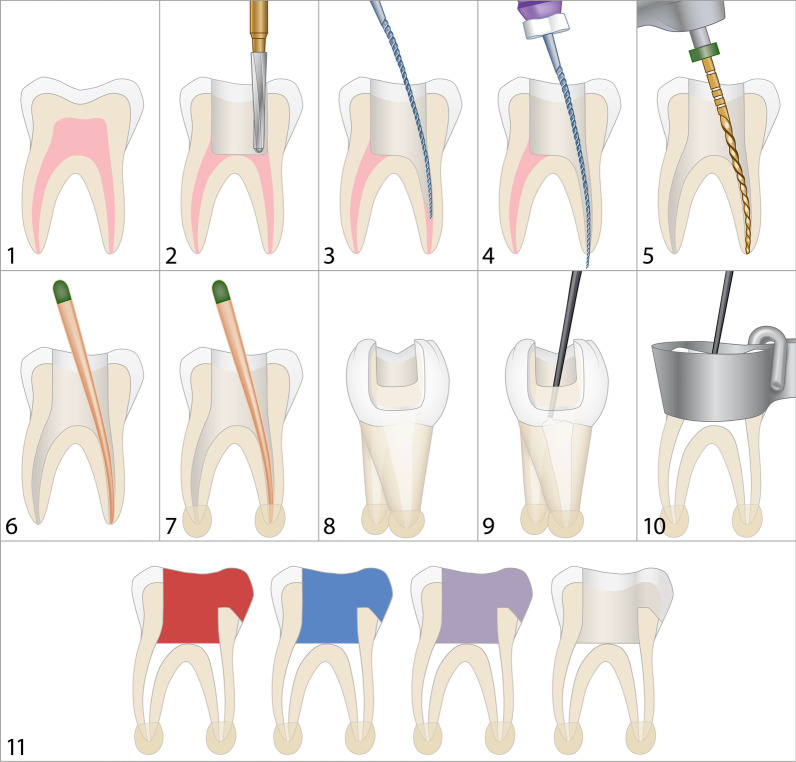
Schematic illustration of the step-by-step procedure of sample preparation

The teeth were randomly divided into four groups of 20 each. The coronal and access cavities of 20 teeth were filled with Cavit W (white), 20 with Cavit (pink) (3 M ESPE, St. Paul, MN, USA) and another 20 with Coltosol F (Coltène/Whaledent AG, Altstätten, Switzerland). One group of 20 teeth without temporary restoration was established as a negative control. A Tofflemire matrix band (KaVo Kerr, Brea CA, USA) was coronally adapted and the coronal as well as the access cavities were completely filled with the specific temporary filling material and moistened with a sponge pellet and a 0.9% NaCl solution (B. Braun Melsungen AG, Melsungen, Germany). All products were used according to the manufacturer’s recommendations. The band matrix was removed after 5 min and the temporary filling materials allowed to set. The coronal and access cavities as well as root canals of the control group were prepared in the same manner; however, no temporary filling material was placed on them. Figure 1 schematically illustrates the step-by-step procedure of sample preparation.

### Storage and tooth labeling

Each tooth was labeled with the corresponding group (I-IV) and number (1–20) . Labeling could be temporary removed when considered necessary during the investigation. Each group was submerged in a separate box containing distilled water at 37 °C (Model 500, Memmert GmbH & Co. KG, Schwabach, Germany) and kept immersed at all times.

### Photographic documentation

The pulp chambers were stained and examined by means of photo-documentation [12] on day 0. Subsequently the temporary filling materials were placed on day 0, the teeth were fixed on a glass slide and photographed from the mesial, distal, buccal, oral and occlusal aspects. The teeth examination was carried out by means of photography under a light microscope (7.5×; Leica M420, Leica Microsystems, Wetzlar, Germany). Each tooth was then examined every 24 hours from day 1 up to day 15. Before being photographed, the teeth were re-dyed through immersion in a freshly mixed 0.25% methylene blue solution, rinsed with distilled water, dried and examined under a light microscope (7.5×). The existence of possible fracture lines in the filling material and on the tooth structure were documented [12]. After day 15, all fillings were gently removed with an ultrasonic instrument (EMS 06 Piezon, EMS Electro Medical Systems GmbH, Munich, Germany) and when necessary with a pear-shaped red diamond size 2 bur (Gebr. Brasseler GmbH and Co KG, Lemgo, Germany). After drying the access cavity, the pulp chamber was stained, examined and photo-documented under magnification (7.5×).

### Evaluation and statistical analysis

A previous sample size calculation was performed using OpenEpi: Open Source Epidemiologic Statistics for Public Health, Version. www.OpenEpi.com. The population size derived from the publication of Tennert et al. [12] with an hypothesized frequency of outcome factor in the population (p) of 20.5%+/− 5 with confidence limits as % of 100 (absolute +/− %)(d) of 5% and a design effect of 1. Due to the confidence level of 90%, a sample size of 73 with a hypothesized error of approximately 10%, the used sample size was set at 80. Two calibrated independent examiners assessed the presence/absence of fractures by means of the obtained photographs and documented the surface of the fracture lines observed in the temporary filling materials, tooth structure or both. In cases where no consensus was reached, a third independent examiner was consulted. Kaplan–Meier curves were calculated to illustrate survival (survival = no fracture) probabilities to evaluate the time when the temporary filling material, tooth structure or both together occurred. Log-rank test was performed in order to assess significant differences between the materials used (Table 1) and investigating differences in the subgroups.

**Table 1:** Total occurrence of tooth structure, temporary filling material and tooth structure and temporary filling material fractures

Group	Tooth structure fracture	Temporary filling material fracture	Tooth and temporary filling material fracture
Cavit (pink)	0	0	0
Cavit W	0	0	0
Coltosol F	8	13	6
Control	0	0	0

*z* = 6.48, *p* < 0.01, sum of ranks: 1884

## Results

**Fig. 2:**
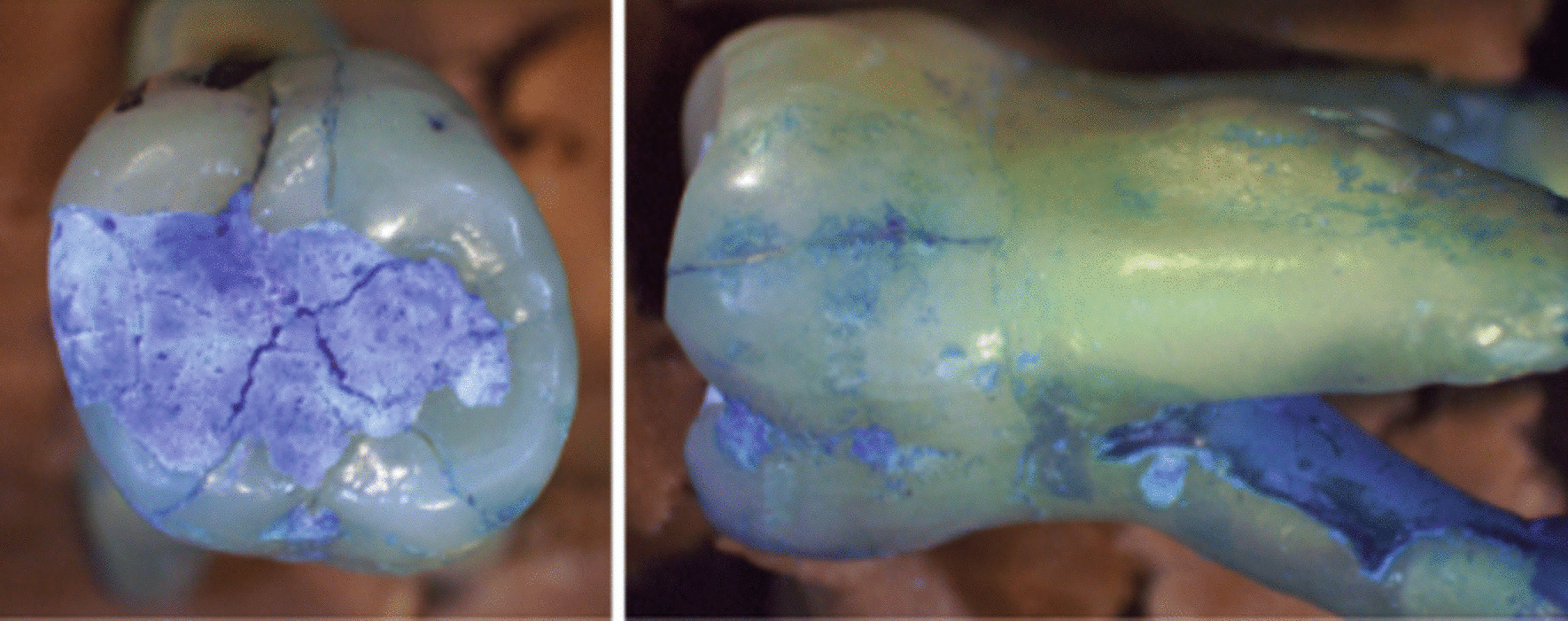
Fractures illustrated in a specimen in the occlusal plane (left) and in the oral plane (right) on day 15 of the experiment

No fractures, neither in the temporary filling materials (TFM), nor in the tooth structure (TS), were observed within the Cavit W, Cavit (pink) and control groups. However, in the Coltosol F group, 13 teeth (65%) showed temporary filling material fractures and eight teeth (40%) showed tooth structure fractures (Fig. 2) at different observation days during the entire examination period of time (Table 1). No fractures were observed in the pulp chamber area in any groups after the temporary filling materials were removed at the end of the observation period.

**Fig. 3:**
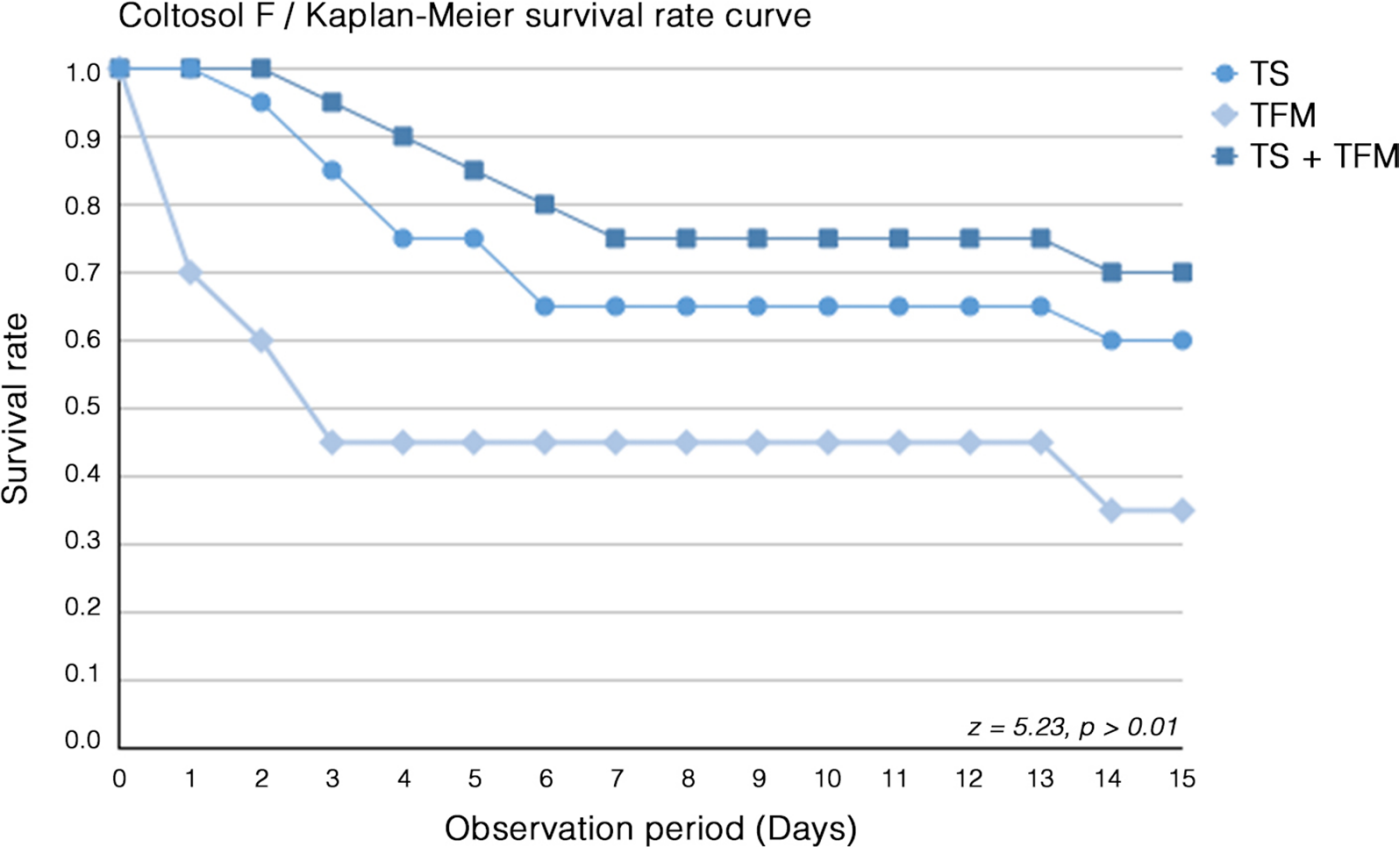
Kaplan–Meier survival curve of specimens filled with Coltosol F without/with fractures of the tooth structure (TS), temporary filling material (TFM) and tooth structure and temporary filling material (TS + TFM) together. The results of the Cavit groups were not calculated since no fractures of any type were observed (n = 20)

In the Coltosol F group, six teeth (30%) showed temporary filling material fractures after 24 h (day 1). During the following 72 h (day 3), another five teeth (45%) yielded fractures within the temporary filling material (Fig. 3). The remaining teeth in this group showed no further temporary filling material fractures until day 14, on which two more temporary filling materials fractured (Fig. 3). Between day 2 and 6, seven (35%) tooth structure fractures also appeared in the Coltosol F group. Only one more tooth structure fracture occurred at day 14 of observation (Fig. 3). In the Coltosol F group, tooth structure and temporary material fractures together appeared constantly between day 3 and 6 in five teeth (25%). No further similar fractures appeared until day 14, on which one more tooth (30%) showed tooth structure and temporary material fractures (Fig. 3). Statistically significant differences (z = 5.23, *p* > 0.01) were observed between; on one side Cavit and Cavit W and on the other side Coltosol F, already after the first (TFM) respectively the fourth day of observation in both (TS + TFM), the temporary filling material and tooth structure sub-groups (Fig. 3).

## Discussion

The design of this investigation conceived the teeth preparation as resembling an endodontic clinical situation as close as possible and assuring that the parameters, control and reproducibility were given at all times. The specimens were selected to be similar in size and form [19, 20] and were stored in distilled water at all times. However, since the periodontal membrane in an in vitro simulation is not achievable in practice, the specimen’s roots were not covered. The tooth and access cavity dimensions and cleaning, shaping and dressing of the root canals were carried out with great attention to detail. The exact composition of the temporary filling materials could be maintained since none of the investigated temporary filling materials had to be mixed. The coronal and access cavities were completely filled with the temporary filling materials. This provided a temporary filling material bulk of at least 3.5 mm, achieving adequate sealing and stability [22]. However, other clinical conditions such as masticatory load can only be simulated to a certain extent in in vitro studies. In a preliminary trial with a thermocycling device, temporary filling material fractures were observed as soon as after 10 cycles, probably due to the intrinsic characteristics of the temporary filling materials. Thus, it was decided not to include thermocycling in this investigation. Two different research parameter distinctions were included. The first one is the distinction made between the coronal cavity and the access cavity. The access cavity comprises solely the removal of the pulp chamber ceiling and probable dimensional extension required in order to be able to obtain a straight access for the root canal instruments. The other parameter distinction that was made is between the three different types of temporary filling material the manufacturers of Cavit produce (Cavit, Cavit W and Cavit G), with their different clinical indications. In our opinion, the name Cavit has caused confusion to a certain extent in the scientific literature since some authors [15, 23–30] unfortunately do not specify which type of Cavit was investigated. It is certainly possible that Cavit (pink) was investigated in these studies; yet, regrettably, it would not be recommendable to draw any conclusions when making a comparison with the results obtained in those investigations. The reason why Cavit (pink) and Cavit W were included in this research is because, according to the manufacturer, these two are the ones to be used as temporary filling material during endodontic treatment [16, 17]. Moreover, in an effort to reduce the possibilities of confusion between these two temporary materials, the authors decided to designate Cavit as Cavit (pink) since it is the color of the material and since it has no further designation as Cavit W and Cavit G do.

A high probability of tooth structure (40%) and temporary filling material (65%) fracture occurred when Coltosol F is used in Class II (OD) cavities of teeth undergoing endodontic treatment. On the other hand, the zinc oxide and zinc phosphate-based materials Cavit (pink) and Cavit W showed no fracture tendencies in either the tooth substance or the temporary filling material itself under the same research parameters during an observation period of 15 days. Previous studies [11, 12, 14] have also shown that tooth structure and temporary filling material fractures appear with a relative high frequency when employing Coltosol F as a result of its hygroscopic expansion and the subsequent degree of water absorption of zinc oxide and zinc phosphate-based temporary filling materials. The information about the temporary filling materials concerning their expansion during their setting process obtained from the internet from manufacturers as well as scientific reports is misleading. According to the incomplete manufacturer’s ingredients information [16, 17, 31] neither Cavit, Cavit W nor Cavit G contain calcium sulphate. However, Naoum and Chandler [7] report that Coltosol F and Cavit (without specifying its type) are zinc oxide and calcium sulphate hygroscopic based materials with a high coefficient of linear expansion resulting from water absorption, which is necessary and intentional for their setting reactions and favorable marginal sealing. Furthermore, due to water absorption, Widerman et al. [32] determined a 14% mean linear setting expansion for Cavit (without specifying its type) and Webber et al. [22] stated that Cavit (type unspecified) has a higher linear expansion coefficient than zinc oxide-eugenol. To the best of our knowledge, on the one hand and according to the manufacturer [18], Coltosol F expands during the setting time; however, more detailed information is not provided. On the other hand, Laustsen el al. [14] report that, according to the manufacturer, Coltosol F has a hygroscopic expansion of 17–20 vol%. These discrepancies could be explained through the different publishing times of different data safety sheets for the investigated products; in any event, they create considerable difficulties in the development of scientific reports.

Tennert et al. [12] compared the mechanical properties impact in teeth with two surfaces (OD) Class II cavities temporary filled with Coltosol F, a combination of Coltosol F and Clearfil as a composite for 14 days. The authors report that 76% of the teeth filled with only Coltosol F showed fractures within the tooth structure and that 16% of them had a non-restorable crown-root vertical fracture. Although the storage conditions and Class II cavity dimensions are similar to the ones of this research, only 40% (n = 8) of the specimens showed a tooth structure fracture in the current investigation. These types of tooth structure fractures can be explained through the stress the hygroscopic material expansion causes in the cavity walls as well as in the temporary material itself. This stress may disappear to a certain degree due to deformation of the cavity walls or temporary filling material fracture, among others [14]. However, this stress release does not prevent a further expansion of the temporary filling material at least until a certain point in time. Therefore, in accordance with Tennert et al. [11], this ongoing expansion could explain that, although fractures in the Coltosol F material itself were observed, fractures of tooth substance occurred as a consequence of further expansion of the material. Another reason for the differences of non-restorable fractured teeth substance between these studies could be due to the biological conditions of the teeth, such as amount of load and age differences of the specimens at the time of implementation [14]. The majority of fractures (35%) in the Coltosol F group in this study were observed within the first six days, suggesting that the water absorption and therefore the expansion of the temporary filling material ceases after the sixth day. This assumption can be supported by the findings of Tennert et al. [11] where no further fractures occurred after five days of observation. However, in contrast to these findings, Laustsen et al. [14] report a continuous intercusp distance increase, which led to a tooth structure fracture, throughout their experiment observation time (20 days). Furthermore, they report that the Coltosol F hygroscopic expansion did appeared in an intermittent manner rather than linearly. These differences could be explained by the fact that the glass-ionomer approximal layer could have influenced the hygroscopic characteristics of Coltosol F [14].

To the best of our knowledge, there has not yet been a study published in which the fracture frequency occurrence of teeth filled with three different temporary filling materials with similar composition have been compared. The fracture rate of teeth temporary filled with Coltosol F has been more often reported than the one for Cavit (pink) and Cavit W. The results of this investigation have shown that zinc oxide and zinc phosphate-based temporary filling materials do not have the same fracture impact on tooth structure per se; yet, it could very well depend on the exact constituent amount of the product itself. Unfortunately, the exact amounts of the ingredients of the products investigated are declared to be trade secrets. Thus, within the limitations of the current in vitro investigation, it has become evident that it is necessary to understand and further investigate whether factors such as the product composition and/or its employment mode induce fractures in the temporary filling material itself and in the tooth structure as well. Limitations of the study are the possible stress-induced influence of the proven expansion of Coltosol F, which could be influenced by the shape of the cavity and the thickness of the walls, which varies in the present study. When a certain limit is reached, the stress-induced deformation causes cracks to appear in the dentin wall, as well as between dentin and enamel, due to the different mechanical properties. Infractures and cracks were not considered in the present study, as the focus was only on detecting clear fractures. It should also be emphasized that the focus of the study was on the occurrence of fractures in the tooth, material or both, and not on the hygroscopic expansion of the materials. When trying to transfer the results of this study to the clinical situation, it is essential to consider the masticatory load and, as an important criterion for the value of the materials, and the consequence of fractures of the tooth and the material, i.e. the permeability of bacteria, which should be further investigated both in vitro and through clinical research.

## Conclusions

Within the limitations of this in vitro study, some considerations are drawn:

- the employment of zinc oxide and zinc sulfate-based materials as temporary filling materials per se does not cause tooth structure fractures in mandibular molars with Class II cavities (OD),
- Coltosol F induced vertical tooth fractures in 40% of the specimens, and.
- specimens temporarily filled with Cavit (pink) and Cavit W did neither show tooth substance nor temporary filling material fractures.

## Data Availability

The datasets generated and/or analyzed during the current study are available from the corresponding author on reasonable request.

## Abbreviations

MOD: Mesial-occlusal-distal
OD: Occlusal-distal
TS: Fractures of the tooth structure
TFM: Temporary filling material
